# Analysis of Nigerians with Apparently Sporadic Parkinson Disease for Mutations in *LRRK2*, *PRKN* and *ATXN3*

**DOI:** 10.1371/journal.pone.0003421

**Published:** 2008-10-17

**Authors:** Njideka Okubadejo, Angela Britton, Cynthia Crews, Rufus Akinyemi, John Hardy, Andrew Singleton, Jose Bras

**Affiliations:** 1 Neurology Unit, Department of Medicine, College of Medicine, University of Lagos, Lagos, Nigeria; 2 Laboratory of Neurogenetics, National Institute of Aging, National Institutes of Health, Bethesda, Maryland, United States of America; 3 Neurology Unit, Department of Medicine, Federal Medical Centre, Abeokuta, Ogun State, Nigeria; 4 Reta Lila Weston Institute and Departments of Molecular Neuroscience and Neurodegenerative Disease, Institute of Neurology, London, United Kingdom; 5 Center for Neuroscience and Cell Biology, University of Coimbra, Coimbra, Portugal; Ohio State University Medical Center, United States of America

## Abstract

Several genetic variations have been associated with Parkinson disease in different populations over the past few years. Although a considerable number of worldwide populations have been screened for these variants, results from Sub-Saharan populations are very scarce in the literature. In the present report we have screened a cohort of Parkinson disease patients (n = 57) and healthy controls (n = 51) from Nigeria for mutations in the genes *PRKN*, *LRRK2* and *ATXN3*. No pathogenic mutations were found in any of the genes. Hence, common pathogenic mutations in these genes, observed in several different populations, are not a frequent cause of Parkinson disease in Nigeria.

## Introduction

The identification of monogenic causes of Parkinson's disease (PD) (MIM #168600) has improved our understanding of the pathogenesis of the disease and promises to facilitate the development of biomarkers to aid early diagnosis and interventions that may slow disease progression or even provide a cure in the future. Mutations in two genes have been linked to autosomal dominant PD; the first, encoding α-synuclein (SNCA; MIM #163890; PARK1) [1] and the second encoding leucine-rich repeat kinase 2 (*LRRK2*; MIM #609007; PARK8) [2], [3]. Mutations in three genes have been linked to autosomal recessive PD: the gene encoding parkin (PRKN; MIM #602544; PARK2)[4], the gene encoding PTEN – induced kinase 1 (PINK1; MIM #608309; PARK6) [5] and the gene encoding DJ-1 (PARK7; MIM #602533; PARK7) [6]. In addition, pathogenic repeat expansion mutations in the genes encoding ataxin-3 (*ATXN3*; MIM #109150) [7] and ataxin-2 (ATXN2; MIM #183090) [8] have been described as autosomal dominant causes of PD.

The frequency of mutations and the risk attributable to polymorphisms in *LRRK2*, *PRKN* and *ATXN3* in patients with sporadic PD has been explored in several studies. Mutations in *LRRK2*, specifically G2019S, appear to be the most common known genetic lesion underlying PD (including the apparently sporadic form), with frequencies as high as 6% in the European population [9] and 41% in North African Arabs [10]. The gene *LRRK2* on chromosome 12q12 encodes a primarily cytoplasmic protein kinase, the function of which has not been clearly elucidated. Parkin is a ubiquitin-protein ligase that maps to chromosome 6q25.2–q27 [14]. Mutations in *PRKN* have been reported as the most frequent cause of autosomal recessive juvenile onset familial PD [11]. However, studies investigating the role of genetic variability at the *PRKN* locus in determining susceptibility to sporadic PD have provided differing results [12], [13]. A wide variety of mutations have been described in *PRKN*, ranging from point mutations to whole exon duplications and deletions. The mechanism by which *PRKN* mutations exert a pathogenic effect is not fully understood, but may involve loss of function and inhibition of parkin's ubiquitin E3 ligase activity and consequent enhancement of neurodegeneration via impaired ubiquitination of substrates [15]. In considering the role of *PRKN* variants in PD, two main notions should be considered. First, heterozygous mutations are fairly common in healthy subjects [13], and second, the presence of an apparent single heterozygous mutation in a PD patient typically should not rule out *PRKN* as the causative gene, since other unscreened mutations may be present [16].

Machado-Joseph disease (MJD/*ATXN3*) is an autosomal dominant progressive neurologic disorder characterized primarily by ataxia, spasticity, and ocular movement abnormalities. The disease is caused by a CAG repeat expansion in the gene *ATXN3*. In 2001, Gwinn-Hardy and colleagues reported a family of sub-Saharan African descent with several individuals displaying parkinsonism suggestive of PD [7]. In this family the *ATXN3* mutation segregated completely with the suggestive PD phenotype. Several cases of PD caused by repeat expansion mutation have been described in African-Americans, and it has been suggested that genetic background may modulate the expressivity of this mutation [17].

In spite of the upsurge in research and publications relating to PD genetics in the past decade, much less is known about the genetics of PD in the African subcontinent, with the majority of publications to date focusing on the North African population [18]. Understanding the genetic associations of PD in Africans will improve our understanding of disease pathogenesis, and improve decision making relating to the usefulness of commercially available predictive genetic tests and preventive and therapeutic interventions that may become available in the future. This study reports the preliminary data from the screening of apparently sporadic cases of PD from West Africa for *LRRK2*, *PRKN* and *ATXN3* mutations.

## Methods

### Population

The study protocol was approved by the Research and Ethics Committee of the Lagos University Teaching Hospital, Lagos, Nigeria. Written informed consent was obtained from all patients and controls. Using a case-control design, 57 unrelated black African PD patients (43 males and 14 females) aged 43 to 80 years and 51 age-matched healthy individuals without a family history of PD or tremor (35 males and 16 females; age range 42 to 87) were recruited from sequentially attending patients at the Neurology Out-patients clinic of the Lagos University Teaching Hospital, Lagos, Nigeria. All patients were evaluated by a neurologist specializing in movement disorders, with keen attention to excluding patients with a possible secondary etiology. The PD cases recruited were those with clinically definite PD only. The inclusion criteria were the presence of all five of: a) at least two of three cardinal signs of tremor, rigidity, bradykinesia (with or without postural or gait abnormality); b) an asymmetric onset; c) no identifiable secondary cause (e.g. repeated stroke, exposure to medications capable of causing PD within 6 months before onset, etc); d) responsiveness to levodopa therapy (applicable to treated patients only); e) absence of signs of more extensive nervous system involvement (e.g. early autonomic features or cognitive impairment within 2 years of onset, otherwise unexplained corticospinal tract dysfunction, and cerebellar signs). All PD cases were evaluated using a standard protocol that included a historical account, neurological examination, Unified Parkinson's Disease Rating Scale (UPDRS) assessment [19], Hoehn and Yahr staging [20], and Folstein's Mini Mental State examination [21]. Control subjects had an abridged neurologic examination to exclude parkinsonism, cognitive impairment, corticospinal tract dysfunction, or any overt neurologic illness. Saliva samples were collected from each case and control using the Oragene kit (DNA Genotek).

The mean age at onset (based on patient's or caregiver's recollection of age at onset of first cardinal symptom of PD) for this group is 58.2±9.1 years (range 40–75). The majority of patients presented no apparent family history for parkinsonism, while nine presented at least one first-degree relative with a history of tremors. This fact may suggest that, for these nine individuals, an autosomal dominant mode of inheritance is possible. Hence, this study will fail to rule out the possibility of mutations in one of the known autosomal dominant PD genes, the *SNCA* gene.

Although all patients are from Nigeria, their specific ethnic origins were as follows: Yoruba – 35 (61.4%), Igbo – 11 (19.3%), and Edo (Ijaw/ Itsekiri/ Urhobo/ other south-south ethnicity) – 11 (19.3%). The ethnic origins of the controls were as follows: Yoruba – 36 (70.6%), Igbo – 9 (17.6%), Edo – 5 (9.8%) and Hausa – 1 (2%).

Further details of cases and controls are presented in Table 1.

**Table 1:** Clinical characteristics of PD cases and controls

*Characteristic*	*PD (n = 57)*	*Controls (n = 51)*
Age at study (mean±SD), years	62.3±9.1	63.7±8.7
Age at onset (mean±SD), years	58.2±9.1	*N/A*
Range of age at onset, years	40–75	*N/A*
Onset before age 50, n (%)	7 (12.3)	*N/A*
Asymmetry of tremors or rigidity at onset, n (%)	57 (100)	*N/A*
Bradykinesia, n (%)	57 (100)	*N/A*
Parkinsonian tremor, n (%)	57 (100)	*N/A*
Rigidity, n (%)	57 (100)	*N/A*
Postural abnormality, n (%)	31 (54.4)	*N/A*
Gait abnormality, n (%)	48 (84.2)	*N/A*
Levodopa responsiveness, n (%)*	54 (94.7)	*N/A*
MMSE [mean±SD (range)]	28.3±2.1 (19–30)	*N/A*
Hoehn & Yahr stage [median (range)]	2 (1–4)	*N/A*
UPDRS - motor subscale [mean±SD (range)]	35.1±12.6 (10–66)	*N/A*
Family history of tremors in a first-degree relative, n (%)	9 (15.8)	*N/A*

* Levodopa responsiveness not applicable in 3 of the 57 who were pre-treatment cases.

### Analysis of mutations

Genomic DNA was extracted from saliva using the Oragene kit (DNA Genotek). *PRKN*, *LRRK2* and *ATXN3* were screened for mutations. For *PRKN*, all exons and intron/exon boundaries were polymerase chain reaction-amplified and sequenced in both directions using BigDye chemistry (Applied Biosystems, Foster City, CA) on an ABI3730xl as previously described [4]. *PRKN* mutations are numbered according to GenBank accession number NP_004553 for the protein (p.) and NM_004562 for the cDNA (c.). *LRRK2* was screened by sequence analysis for the most common mutations occurring in exons 31 and 41 as previously described [9]. *ATXN3* pathogenic repeat expansion size was assessed in all samples using methods described previously by us [17].

### Copy number analysis

In addition to sequencing *PRKN*, we screened *PRKN* for copy number mutations in two samples carrying heterozygous mutations not found in controls (p.P153R; c.C458G and p.R334H; c.G1001A), using the Illumina Infinium HumanHap550 BeadChips (version 3; Illumina Inc, San Diego, CA, USA) as previously described [22]. Copy number analysis by genotyping was done according to the manufacturer's protocol (Illumina Inc.) using 750 ng of genomic DNA. Data was analysed with BeadStudio v3 (Illumina Inc.) using the Human Genome build 35. Two metrics were visualized using this tool, B allele frequency and log R ratio. The former is the theta value for an individual SNP, which gives an estimate of the proportion of times an individual allele at each polymorphism is called A or B. The log R ratio is the log2 ratio of the observed normalized R value for the SNP divided by the expected normalized R value for the SNPs theta value. An R above 1 is indicative of an increase in copy number, and values below 1 suggest a deletion. We have shown previously that this is a reliable method for detecting large genomic copy number mutation in *PRKN* [22].

## Results

We did not find any variants in exons 31 and 41 of *LRRK2*. Likewise, the screening for the *ATXN3* repeats revealed that no samples contained pathogenic expansions. The *PRKN* gene yielded several variants, some new and others previously described. With the exception of the V380L and S167N polymorphisms, no other missense homozygous variants were found. All the *PRKN* variants are shown in Table 2. Additionally, samples that showed only one heterozygous mutation in *PRKN* that was not present in controls were screened for copy number variants in this gene, in order to assess if these were in fact compound heterozygous for one point and one genomic copy number mutation. Genomic copy number analysis did not detect any mutations in these samples.

**Table 2:** *PARK2* gene variants in PD cases and controls

Gene	Heterozygous variants	*Parkinson's disease*	*Control group*
		N (%)	N (%)
PARK2	p.E16E; c.G48T	3 (5.3)	1 (2.0)
	p.P37P; c.G111A	8 (14.0)	6 (11.8)
	p.A46T; c.G136A	1 (1.8)	0
	p.P153R; c.C458G	0	1 (2.0)
	p.S167N; c.G500A	4 (7.0)	4 (7.8)
	p.M192L; c.A574G	7 (12.3)	6 (11.8)
	p.L261L; c.A783G	13 (22.8)	14 (27.4)
	p.G319G; c.T957C	5 (8.8)	4 (7.8)
	p.R334H; c.G1001A	1 (1.8)	0
	p.V380L; c.G1138A	2 (3.5)	3 (5.9)
	**Homozygous variants**		
	p.P37P; c.G111A	1 (1.8)	3 (5.9)
	p.S167N; c.G500A	1 (1.8)	0
	p.L261L; c.A783G	1 (1.8)	3 (5.9)
	p.V380L; c.G1138A	1 (1.8)	1 (2.0)

## Discussion

This is the first study screening a sub-Saharan African cohort of apparently sporadic PD cases for mutations in genes commonly associated with PD. The results from this study are of clear importance not only for Nigerian PD patients, but also because they shed light on the genetic background associated with PD in the African population. It should be noted that the number of individuals included in this preliminary report is clearly small, and thus, definite conclusions about frequencies of variants are difficult to achieve.

We have performed a screening for the most common autosomal recessive variants in three genes associated with PD (*LRRK2*, *PRKN* and *ATXN3*). We decided not to screen for mutations in the genes *PINK1*, *DJ-1* and *ATXN2* given the low frequency of mutations in these genes in worldwide populations. Moreover, we did not screen *SNCA* as mutations in this gene are not only rare but they are also associated with an autosomal dominant mode of inheritance. Given that the majority of our patients have no affected family members with any form of parkinsonism, mutations in *SNCA* would be unlikely to occur in our cohort. Mutations in the *LRRK2* are the most common cause of PD in several populations, including populations of Northern-African ancestry. In particular, Lesage and colleagues found a high frequency (41%) of the G2019S mutation on exon 41 in a study of North African Arabs that included both familial and apparently sporadic PD cases [10]. Thus, it would be interesting to determine if there is a similarly high frequency of *LRRK2* G2019S mutations in other geographically and ethnically distinct parts of Africa. However, we did not find any mutations in either exons 31 and 41 of *LRRK2* in our cohort, suggesting that mutations in these domains of *LRRK2* are not a common cause of PD in sub-Saharan populations. A recent study performed a comprehensive analysis of the entire coding region of *LRRK2* in a large cohort of American PD cases and healthy controls [23]. Of the seven mutations found to be segregating with disease, five were in either exon 31 or exon 41, indicating these as clear mutational hotspots. The noteworthy difference in mutation frequency among populations from the same continent is, in all probability, due to the occurrence of the founding G2019S mutation event happening after human populations moved out of sub-Saharan Africa as this is most consistent with the dating of this mutational event [24], [25].

Parkinsonism due to *ATXN3* repeat expansion mutation has been previously described in one single large African descent family. We failed to find any samples harboring the increased repeat expansion size. This result suggests that *ATXN3* repeat expansion mutations are not a frequent cause of parkinsonism in this population.

Even though a considerable number of *PRKN* mutations are dosage mutations, the majority are sequence variants, hence, we decided to perform the initial screen of our cohort only for these variants. Subsequently, we performed gene dosage analysis in two samples as previously detailed. Again, our study did not identify any pathogenic mutations in *PRKN* in our subset of PD patients. We found several heterozygous variants both in patients and controls.,Two of the variants are novel and present only in PD cases (p.R334H and p.A46T). Given the fact that these are novel variants, we ran the analysis software SIFT (available at http://blocks.fhcrc.org/sift/) [26] in order to have some insight into the potential effects of these variants. The p.A46T was predicted by the software to potentially affect protein function, whereas the p.R334H was predicted to have no functional effect. However, these results are merely based on a similarity score in comparison to other proteins, and hence it must be stressed that this is not true functional data for these variants. Nonetheless, in the absence of a second mutation, these cannot be described as pathogenic, thus we decided to classify these as variants of unknown pathogenicity. It should be noted that for these two samples, an additional screen for gene dosage mutations was performed using the Illumina BeadChips. In addition, one homozygous variant was found only in the PD group (p.S167N; c.G500A). However, this has previously been classified as a polymorphism [12], [13].

All populations showed polymorphisms with varying frequencies. Three variants p.A46T, p.P153R and p.R334H were found in a single sample each. Variant p.A46T was present in one PD sample from Igbo, p.P153R was found in a Yoruban sample, and p.R334H was present in a sample with Edo background. Two individuals, both from the same ethnic region (Edo), presented with two missense variants each (p.M129L and p.S167N; p.M129L and p.V380L). Although the present study cannot completely rule out that these compound hetererozygous events could potentially be pathogenic, two facts suggest otherwise: 1) in each case the second variant is a well known and described SNP; 2) one control sample presented the same combination of two of the variants (p.M129L and p.V380L).

We report the first genetic screening for PD genes in a sub-Saharan population. We found no pathogenic mutations in the genes most commonly known to cause PD in European North American, or North African populations. Although the cohort studied is clearly small and definite conclusions regarding frequencies are unachievable, a trend for different genetic basis of PD in this sub-Saharan population is, in our opinion, noteworthy. Two main caveats are present in this work: gene dosage mutations in *PRKN* were only screened for in two samples and only exons 31 and 41 of *LRRK2* were sequenced. Nevertheless, the aim of this study was to ascertain a preliminary frequency of the most common variants known to cause PD in a sub-Saharan population. We report here that the most common variant associated with PD in several world-wide populations, the p.G2019S mutation in *LRRK2*, is not overrepresented in this Nigerian population of PD patients; similarly, sequence variants in *PRKN*, which represent a significant proportion of *PRKN* mutations underlying PD, are also not significantly present in the studied cohort. It is thus likely that the differences in the genetic background of these populations mean that other genes or different variants are underlying the disease. Therefore, a search for these is clearly warranted, since they will, in all probability, shed more light on different pathways leading to PD.
